# Occupational class, gender, mental distress and use of psychotherapy: Sanna Selinheimo

**DOI:** 10.1093/eurpub/ckac129.685

**Published:** 2022-10-25

**Authors:** L Kokkinen, K Gluschkoff, J Kausto, S Selinheimo, A Väänänen

**Affiliations:** 1 Faculty of Social Sciences, Tampere University, Tampere, Finland; 2 Finnish Institute of Occupational Health, Helsinki, Finland

## Abstract

**Introduction:**

Previous studies have shown the effects of occupational class and gender on mental distress and use of psychotherapy. However, less is known about the mental distress-based use of psychotherapy in different occupational classes.

**Objectives:**

The aim of our study is to show how the prevalence of mental distress and the use of long-term psychotherapy correlates in different occupational classes by gender.

**Methods:**

Data were drawn from the Rise of Mental Vulnerability Study (psychotherapy) and FinHealth 2017 Study (mental distress). Adjusting for age, we calculated GHQ caseness, psychotherapy use rate, and the ratio between GHQ caseness and psychotherapy use rate in three occupational classes (upper non-manual employees, lower non-manual employees, and manual workers) separately for men and women. Age-adjustment was performed through regression analysis by using model generated predicted values of GHQ caseness and psychotherapy use rate at the average sample age of 40.

**Results:**

In the group of upper non-manual men there were 10 persons with severe mental distress for every single person having used psychotherapy. For lower non-manual men and manual male workers the numbers were 14 and 31, respectively. In the group of upper non-manual women there were six persons with severe mental distress for every person having used psychotherapy. For lower non-manual women and manual female workers these numbers were nine and 18, respectively.

**Conclusions:**

We found differences in the mental distress-based use of state-subsidized long-term psychotherapy between occupational classes. For upper non-manual workers, the use of therapy best meets their mental distress. The opposite is true for manual workers. We also found differences between men and women, but these findings should be confirmed with larger datasets.

